# A pantropical assessment of deforestation caused by industrial mining

**DOI:** 10.1073/pnas.2118273119

**Published:** 2022-09-12

**Authors:** Stefan Giljum, Victor Maus, Nikolas Kuschnig, Sebastian Luckeneder, Michael Tost, Laura J. Sonter, Anthony J. Bebbington

**Affiliations:** ^a^Institute for Ecological Economics, Vienna University of Economics and Business, 1020 Vienna, Austria;; ^b^Advancing Systems Analysis Program, International Institute for Applied Systems Analysis, A-2361 Laxenburg, Austria;; ^c^Chair of Mining Engineering and Mineral Economics, Montanuniversität Leoben, 8700 Leoben, Austria;; ^d^School of Earth and Environmental Sciences, The University of Queensland, St. Lucia, QLD 4072, Australia;; ^e^Centre for Biodiversity and Conservation Science, The University of Queensland, St. Lucia, QLD 4072, Australia;; ^f^Graduate School of Geography, Clark University, Worcester, MA 01610

**Keywords:** deforestation, indirect effects, land-use change, large-scale mining, tropical forests

## Abstract

Growing demand for minerals continues to drive deforestation worldwide. Tropical forests are particularly vulnerable to the environmental impacts of mining and mineral processing. Many local- to regional-scale studies document extensive, long-lasting impacts of mining on biodiversity and ecosystem services. However, the full scope of deforestation induced by industrial mining across the tropics is yet unknown. Here, we present a biome-wide assessment to show where industrial mine expansion has caused the most deforestation from 2000 to 2019. We find that 3,264 km2 of forest was directly lost due to industrial mining, with 80% occurring in only four countries: Indonesia, Brazil, Ghana, and Suriname. Additionally, controlling for other nonmining determinants of deforestation, we find that mining caused indirect forest loss in two-thirds of the investigated countries. Our results illustrate significant yet unevenly distributed and often unmanaged impacts on these biodiverse ecosystems. Impact assessments and mitigation plans of industrial mining activities must address direct and indirect impacts to support conservation of the world’s tropical forests.

## Significance

Driven by rapidly increasing demand for mineral resources, both industrial mining and artisanal mining are intensifying across the tropical biome. A number of regional studies have analyzed mining-induced deforestation, but scope and patterns across all tropical countries have not yet been investigated. Focusing on industrial mining, we use geospatial data to quantify direct forest loss within mining sites in 26 countries. We also perform a statistical assessment to test whether industrial mining drives indirect deforestation in the mine surroundings. We show that direct deforestation concentrates only in a few countries, while industrial mining causes indirect deforestation in two-thirds of tropical countries. In order to preserve tropical forests, direct and indirect deforestation impacts of mining projects should be fully considered.

Driven by rising affluence and surging demand for minerals for consumer products, infrastructure, and energy transition technologies, global mining activities expanded at an unprecedented pace in the past 20 y (1, 2). Today, mines worldwide extract double the amount of raw materials compared with the year 2000 (3, 4), with the trend expected to continue in the coming decades (5, 6). Growing raw material extraction causes a wide range of environmental impacts, including disturbance of ecosystems and protected areas and biodiversity loss as well as water scarcity and pollution (7–10). Resource-extracting regions face extensive land-use changes due to the expansion of mining activities and related infrastructure, often accompanied by deforestation (11–16). The tropical biome is particularly vulnerable to mining-related impacts (12, 17). The high density of wetlands and rivers increases the probability of pollution of water bodies by toxic substances, such as acids used as solvents to separate the metal content from the mined crude ore (18). Tropical rainforests are also a major carbon storage (19). Mining-related deforestation thus destroys carbon storage capacities, with implications for global climate stability.

Compared with other causes of tropical deforestation, such as crop production or livestock farming, mining so far is a minor driver, but its growing importance has been emphasized in various case studies in the Amazon region (17, 20, 21) and in India (16). The significance and geographical pattern of deforestation induced by mining across tropical forests worldwide are yet unknown. This is problematic, as current estimations on the overall effects of mining on forests rely on broad extrapolations of case study results (22), ignoring differences between countries. Further, this gap hinders an efficient allocation of global conservation investment, which requires knowledge about where tropical mining causes the most deforestation impacts. In this study, we provide an investigation of deforestation impacts induced by industrial mining operations across 26 countries located in tropical wet and dry forests. The selected countries together cover 76.7% of total deforestation observed in tropical forest biomes in the period from 2000 to 2019 (23).

**Fig. 1.**
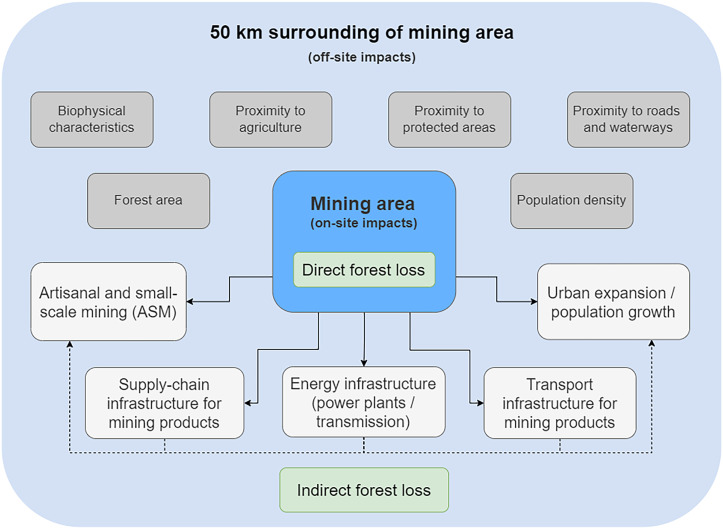
Conceptual framework of direct and indirect forest loss related to industrial mining activities. Direct deforestation is quantified as forest loss within mining areas. Infrastructure, settlements, and artisanal and small-scale mining (white boxes) are conceptualized as effects causing indirect deforestation induced by mining activities in an area of 50 km surrounding industrial mines. Gray boxes indicate control variables in the statistical assessment. *SI Appendix*, Fig. S3 shows a more extensive illustration of indirect deforestation pathways.

Our assessment framework considers both direct and indirect deforestation impacts of industrial mining (Fig. 1). Direct deforestation occurs within the mining area itself through establishing or expanding extraction sites, tailing storage facilities, waste rock dumps, and on-site processing facilities and roads. In addition to quantifying direct deforestation within mining areas, we set up a statistical model to assess whether mining induces indirect deforestation in its surroundings. Indirect deforestation occurs outside areas designated for mining and emerges through various pathways. For example, mineral extraction and processing require large amounts of energy, demanding infrastructure for energy generation. Building up infrastructure for mineral processing, storage, and transport is another pathway leading to indirect deforestation (12). Expansion of mining sites may also lead to in-migration and growth of settlements in the surrounding areas, creating new agricultural land and pastureland with impacts on forest loss (24). With this study, we explore whether these indirect deforestation effects of mining can be found in countries across the tropical biome. These indirect effects have hardly been quantified so far. One study of the Brazilian Amazon showed that mining induces deforestation up to 70 km from mining leases and that indirect deforestation is 12-fold higher than forest loss within mining leases (9).

We focus our study on the direct and indirect deforestation impacts of industrial mining. There is ample evidence that artisanal and small-scale mining (ASM), both legal and illegal, has had a growing impact on deforestation in many tropical mining countries in recent years (10, 17, 22, 25). Some datasets on ASM locations have been presented, for example, for selected African countries (26–28). However, in contrast to industrial mining, no database with a worldwide scale exists that would allow for considering ASM locations and extents consistently in a spatial statistical assessment covering the whole tropical biome. Further, the presence of the ASM sector can ebb and grow much more rapidly than that of larger-scale industrial mining. Mining areas are thus often difficult to identify after they have been abandoned, as they are characterized by a mix of bare ground, water, and remaining or new vegetation (25). This poses additional challenges when trying to quantify the extent of ASM over a time period of 20 y. Due to current inconsistencies and limitations of ASM-related data, we are not able to address the expanding ASM sector as a direct driver of deforestation and thus, to capture the overall deforestation impacts of mining. Instead, we only cover those ASM activities that are an indirect effect of industrial mines given that formal large-scale mining often opens up new exploration areas, attracting informal ASM activities to follow (29).

Our comprehensive assessment of industrial mining in tropical countries reveals that most direct deforestation between 2000 and 2019 occurred in only four countries (Indonesia, Brazil, Ghana, and Suriname). Indirect deforestation impacts of industrial mining activities are found in 18 of 26 investigated countries across Latin America, Africa, and Asia. Particularly strong indirect impacts are observed in the deforestation hot-spot countries Indonesia and Brazil. The scientific evidence underlines the importance of considering both the direct and indirect impacts in environmental assessments of mining activities and related infrastructure planning in countries with tropical forests (30).

## Results

### Direct Deforestation within Mining Areas.

**Fig. 2.**
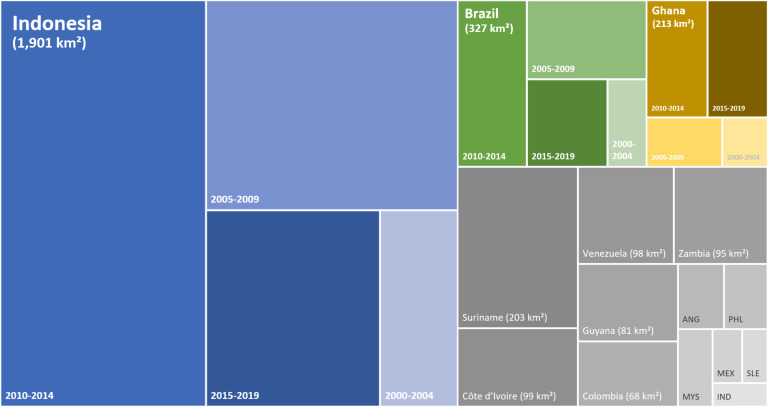
Direct tropical forest loss due to industrial mining from 2000 to 2019. The tree map illustrates the top 15 countries with the highest absolute deforestation by industrial mining, together accounting for 98% of direct deforestation across all 26 investigated countries. For the top three countries of Indonesia, Brazil, and Ghana, total forest loss is presented in 5-y periods. An illustration of direct deforestation per time period is in *SI Appendix*, Fig. S2. ANG, Angola; IND, India; MEX, Mexico; MYS, Malaysia; PHL, the Philippines; SLE, Sierra Leone.

Investigated mining areas covered 11,467 km^2^ of land that included 7,019 km^2^ of tropical forest in 2000. By 2019, 3,264 km^2^ (46.5%) of these forest areas were directly lost to industrial mine expansion (*SI Appendix*, Table S2). With 1,901 km^2^ of deforested area, Indonesia was by far the most affected country, accounting for 58.2% of direct forest loss by mining across all 26 investigated countries (Fig. 2). Mine expansion in East Kalimantan on the island of Borneo for coal production (31–33) was the main factor behind this development in Indonesia. Deforestation within Brazil’s mining areas located in tropical forest biomes extended over 327 km^2^ since 2000, representing 10% of the direct tropical forest loss by mining across all 26 countries analyzed here. Note that direct mining-related deforestation for Brazil is only around a third of the impact reported for the Brazilian Amazon in an earlier study (9) that investigated forest loss in mining concession areas instead of the actual mining areas investigated in this study. Ghana (213 km^2^, 6.5%), Suriname (203 km^2^, 6.2%), and Côte d’Ivoire (99 km^2^, 3%) follow as the countries with the highest direct forest loss. All other countries together made up 16% of tropical forest loss by mining observed across the 26 countries (*SI Appendix*, Table S3).

We split up forest loss within mining areas into four 5-y periods and find that direct deforestation by mining was significantly higher in the time period after 2010, with 65% of forest loss since 2000 occurring in the past 10 y. With 45% of direct forest loss since 2000, the highest mining-related deforestation rate in Indonesia was observed for the period 2010 to 2014. Also, Brazil’s deforestation peak occurred in those 5 y (36% of total forest loss), while direct forest loss in mining areas slowed down after 2015 (*SI Appendix*, Fig. S2 and Table S3).

Compared with other land-intensive activities, such as the production of soybeans and palm oil or cattle farming, the direct deforestation impact of mining is small in many countries with tropical forest biomes. The 1,901 km^2^ of deforestation within Indonesia’s mining areas contributed 0.7% to the total forest loss of 267,594 km^2^ since the year 2000 (23). In Brazil, on-site deforestation had a share of 0.06%. However, we also found that mining played a larger role in tropical deforestation for some countries, even though the absolute mine expansion was small compared with that in Brazil and Indonesia. We found the highest shares for Suriname (11%) and Guyana (4%). Both countries observed low overall deforestation numbers in the past 20 y (1,842 and 2,051 km^2^, respectively), and a significant share was related to forest loss within mining areas, with bauxite and gold being the main extracted commodities (34).

### Indirect Deforestation Induced by Mining.

In addition to direct forest loss, we find strong evidence that mining induces indirect deforestation outside areas designated for mining activities. In 18 of the 26 investigated countries, deforestation rates are higher close to the actual mining areas than areas farther away than 50 km, even when controlling for other known determinants of tropical deforestation. The presence of this indirect impact on deforestation is estimated using the distance to the closest mine as a regressor. A negative coefficient with high statistical significance indicates that mining drives off-site deforestation and that deforestation decreases with growing distance from mines (Table 1).

**Table 1.** Indirect deforestation effects induced by industrial mining in 26 tropical countries

Country	*δ*	SE	*N*	*R* ^2^
Angola	–0.208	0.027	67,993	0.08
Brazil	–0.306	0.004	2,273,696	0.34
Côte d’Ivoire	0.086	0.004	72,565	0.98
DRC	–0.141	0.013	280,658	0.18
Colombia	–0.321	0.021	38,125	0.34
Gabon	–1.965	0.122	6,588	0.13
Ghana	–0.037	0.011	36,982	0.95
Guinea	0.371	0.015	72,633	0.81
Guatemala	–0.073	0.079	8,604	0.29
Guyana	–0.551	0.013	163,572	0.11
Honduras	–0.178	0.047	3,325	0.30
Indonesia	–0.227	0.006	621,704	0.46
India	0.015	0.002	846,387	0.37
Liberia	–0.279	0.022	41,739	0.43
Mexico	–0.082	0.012	152,740	0.37
Mozambique	0.822	0.022	48,977	0.56
Malaysia	–0.036	0.019	79,174	0.34
Nicaragua	–0.493	0.044	20,153	0.62
The Philippines	–0.253	0.018	57,652	0.34
Papua New Guinea	–0.241	0.025	63,933	0.21
Sierra Leone	–0.079	0.022	17,961	0.50
Suriname	–0.874	0.020	105,970	0.10
Thailand	0.349	0.015	77,423	0.53
Tanzania	0.090	0.014	69,522	0.67
Venezuela	–0.168	0.019	187,993	0.17
Zambia	–1.373	0.023	96,802	0.29

The dependent variable is log-transformed accumulated forest loss area between 2000 and 2019, and the explanatory of interest is the log-transformed distance to the nearest mine. The ordinary least squares coefficient *δ* is the associated elasticity between forest loss and the distance to the nearest mine, and *N* is the number of observations. *SI Appendix*, Table S1 shows the control variables.

In Brazil and Indonesia, we find high statistical significance (i.e., very small SEs) for mining driving deforestation in the surrounding areas up to 50 km outside the mining polygons. Reducing the distance to the nearest mine in Brazil by 10% (for example, from 10 to 9 km) while holding all other variables constant implies an average increase of deforestation by 3% (*N*/number of grid cells = 2.27 million; *P* < 0.001) (Table 1). For Indonesia, this value is 2.3% (*n* = 621, 704; *P* < 0.001). We can illustrate the absolute effect on deforested areas with a scenario simulation. For example, assuming that all mines in Indonesia expand their borders by 100 m, this would induce an additional deforestation between 194 and 215 km^2^. In Brazil, the same 100-m expansion would lead to 147 to 154 km^2^ of additional forest loss (*SI Appendix*, Table S8). Assuming an alternative scenario of an expansion by 1 km instead of 100 m would imply a linear scaling-up of the impacts by a factor of 10.

The significance of the distance to mines in comparison with other major deforestation drivers can also be illustrated by comparing the coefficient of the distance to a mine with the control variable for agricultural production (distance to the nearest agricultural area). In Brazil, the indirect deforestation coefficient of a mine is approximately a third of the effect caused by the proximity of agricultural areas. For Indonesia, the mine-related coefficient is around 29% of the effect of the distance to agriculture (*SI Appendix*, Table S4).

Apart from Indonesia and Brazil, the statistical relation of mines causing indirect deforestation can also be observed for many other tropical countries (Table 1). These include Guyana (5.5% increase in deforestation when moving 10% closer to a mine), Colombia (3.2%), the Philippines (2.5%), Papua New Guinea (2.4%), Democratic Republic of the Congo (DRC; 1.4%), and Ghana (0.3%). For some mining countries in Africa, the indirect deforestation impact of mining is particularly strong, such as in Gabon (19%) and Zambia (13%).

For eight countries, our model does not identify the presence of a mine as a notable driver of deforestation in the surrounding areas. For Guatemala and Malaysia, model results are not statistically significant. In some countries, our model suggests that deforestation decreases with closeness to mines, indicated by a positive coefficient for that variable (Table 1). For example, for Thailand, approaching a mine by 10% would lead to reduced deforestation of 3.5%. In that country, mining areas are found in the north, whereas major deforestation areas are located in the south. The latter areas are thus not impacted by mines in Thailand but possibly, are impacted by mining activities in the north of Malaysia. However, as we calculate one average effect for each country and cut off our assessment at country borders, we neglect possible cross-country drivers.

**Fig. 3.**
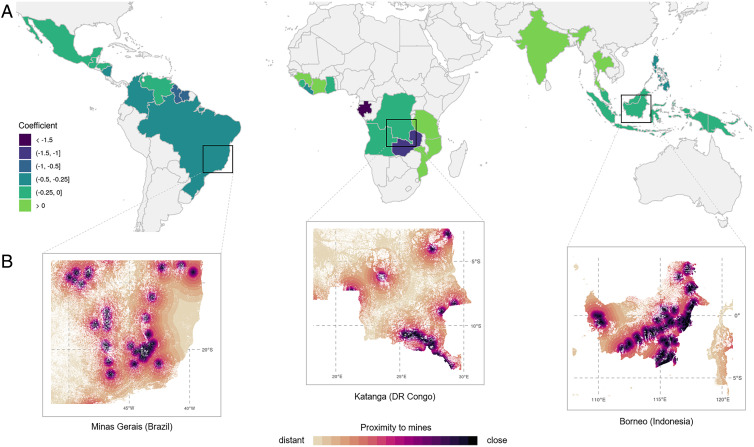
Visual representation of indirect deforestation induced by industrial mining outside mining areas. (*A*) The national coefficient of distance to mine (log) across 26 investigated countries. (*B*) Granular representation of the national coefficient in three selected mining areas. The closer a grid cell is located near a mining site, the higher is the share of mining-induced deforestation. DR, Democratic Republic.

Fig. 3 provides a visual representation of the statistical results. The maps (Fig. 3*A*) show the coefficient for mining-induced deforestation in all 26 investigated mining countries with tropical forests. The impacts of industrial mining on forest loss can also be illustrated on a spatially explicit level by considering the distance of each grid cell to the nearest mine (Fig. 3*B*). These maps for three selected mining regions in Brazil, the DRC, and Indonesia thus illustrate the importance of mining-induced deforestation in total forest loss in each grid cell. White areas indicate that no forest loss has been observed since 2000. The importance of forest loss due to industrial mining is clearly visible in the state of Minas Gerais in Brazil, where iron ore and gold are particularly mined in the “Iron Quadrangle” in the south. The indirect deforestation induced by mining in the Central African Copperbelt, stretching between Zambia and the DRC, is illustrated in the second example. As a third case, we show the Indonesian mining regions on the island of Borneo, where coal, nickel, and tin mining significantly expanded since the year 2000.

## Discussion

This study has provided an assessment of deforestation caused by industrial mining in countries across the tropical biome. Our results show that the direct impacts of mining on tropical forests have been concentrated in only a few countries, with Indonesia, Brazil, and Ghana being the most heavily affected. In Indonesia, the highest deforestation rates were observed for the years 2010 to 2014, a period that was marked by a doubling of coal production volumes, particularly driven by demand from China and India. A fragmented and opaque governance system for issuing new coal extraction licenses facilitated this development. Institutional reforms after 2014 implemented caps on coal extraction growth rates (35), which also slowed down direct deforestation. Also in Brazil, forest loss within mining areas decreased after 2014. Declining global commodity prices and an economic crisis in Brazil after 2014 are among the explanatory factors for that temporal pattern. We also found that mining played a large role in the deforestation trajectories in other countries with relatively less absolute forest loss (e.g., 11% of deforestation in Suriname between 2000 and 2019 was directly caused by mining).

Further, we revealed that industrial mining indirectly drives deforestation in the surroundings outside mining areas. Indirect deforestation effects caused by industrial mining are most considerable for Brazil and Indonesia, but these effects can be observed in more than two-thirds of the investigated countries. Against the background of rapidly increasing global demand for mineral resources, e.g., for housing and transport infrastructure (36) or green energy technologies (37), our results emphasize important yet unevenly distributed and largely unmanaged future threats to tropical forests.

### Limitations of the Approach and Further Developments.

Our approach has several limitations that need to be addressed in further research. First, our study focused on industrial mining activities only and conceptualized parts of ASM as a potential indirect effect of industrial mines. Important tropical mining countries, such as Peru and Bolivia, have been excluded altogether, as too few industrial mines are located in tropical forests and direct forest loss caused by ASM has not yet been comprehensively quantified. Further, the relation between large- and small-scale mining is often complex; formal mining can open up new areas, attracting informal ASM activities to follow, or ASM activities start exploiting mineral resources, with industrial mining players later moving there (29). A precondition for proper conceptualizing ASM vis-a-vis industrial mining is the establishment of an international monitoring system of the geographical extents and developments of ASM, potentially as a part of already established ASM data platforms, such as Delve, coinitiated by the World Bank (https://delvedatabase.org/).

Second, regarding indirect deforestation effects, our statistical analysis addressed the question of the causal relation between mining and deforestation in its surroundings. Equally important is the quantification of the actual indirectly deforested areas driven by expanding mining activities (9) and investigating the factors that explain the magnitude of these indirect effects. To accurately disentangle causal pathways and measure specific impacts, more comprehensive data that allow for accounting for local circumstances as well as flexible methods that offer insights beyond average effects (38) are needed.

Third, our study provided an overview assessment across tropical mining countries but could not deliver detailed insights into why differences across countries regarding the scope of indirect effects can be observed. For this, case study countries need to be selected, and additional datasets need to be incorporated that allow for time tagging of key variables: for example, when a specific mine has started its operation or when infrastructure projects have been realized.

Fourth, a key improvement area is to assess variations in deforestation patterns caused by mining of different commodities, as deforestation impacts will differ, for example, between deep open pit mines, which often characterize copper extraction, and near-surface mining in the case of nickel laterite mines. Further, price differences between commodities could impact the dynamics between industrial mining and ASM and thus, influence the attribution of indirect deforestation to industrial sites. Against the rapidly growing demands in particular for metals for renewable energy and e-mobility technologies, such commodity-specific information is key for designing government and industry policies.

Finally, mine rehabilitation and reforestation efforts should be considered in future geospatial assessments of the impacts of mining activities on forest ecosystems.

### Implications for Policy and Industry.

For policy makers and mining companies operating in tropical countries, our results suggest the Environmental Impact Assessments and licensing procedures for new mining projects should also consider potential impacts outside the actual mining extents. According to an analysis by the World Bank (22), there is a general deficit of impact assessments that take the off-site impacts of mining on forest ecosystems into account. Studies analyzing the indirect effects of mining on forest loss in quantitative terms could pave the way for a more explicit requirement to assess intact forests and biodiversity within a defined distance around new mining projects. Applications for new mining concessions should also not be considered in isolation—in particular, if they include additional economic sectors, such as agriculture—but should take their potential cumulative impact on forest loss into account (39).

However, given the current political context in many tropical mining countries, like Brazil and Indonesia (13, 40), it is questionable whether proenvironment policies will be implemented in the near future. Other actors, including conservation organizations, multilateral organizations, and industry groups, will therefore play a key role in setting stricter environmental standards. Although companies in the extractive sectors have started to manage direct deforestation impacts of mining activities, only a few examples consider indirect impacts on deforestation (15). Initiatives, such as the Proteus partnership between the United Nations Environment Programme’s World Conservation Monitoring Centre and leading companies in fossil fuel and metal extraction (https://www.proteuspartners.org/), provide a forum to increase collaboration between companies to address potential cumulative impacts of their mining activities (22).

Best-practice approaches to mitigate mining impacts on forests are promoted by private sector organizations, such as the International Council on Mining and Metals (ICMM) (41). However, off-site impacts are not addressed appropriately yet. ICMM also has a requirement in place for its member companies not to explore or mine in World Heritage areas listed by the International Union for Conservation of Nature (IUCN). Opportunity exists to expand these commitments to avoid impacts on other biodiverse places.

At the international level, policy initiatives that aim at raising transparency and due diligence along global supply chains could also play an increasingly significant role in managing environmental impacts of mining. For example, in 2019, the European Union adopted a strategy to protect the world’s forests by reducing its global land footprint and minimizing its consumption of products linked to deforestation (42). Another example is Section 1502 of the US Dodd–Frank Wall Street Reform and Consumer Protection Act (commonly referred to as the Dodd–Frank Act) that came into force in 2010, which requires certain due diligence actions from companies importing gold, tin, tungsten, and tantalum from the DRC or its neighboring countries. In the future, policy incentives could also be set regarding environmental aspects: for example, allowing market access only to companies that monitor and limit the deforestation impacts of their products. One key requirement to limit losses of tropical forests due to mining is the development and implementation of monitoring programs. Satellite-based data systems on mining activities and deforestation, as employed in this study, could form the starting point for developing monitoring systems that allow for a regular and consistent identification of deforestation linked to the expansion of specific mines. Such information is also a precondition to set up supply chain initiatives in the private sector to reduce deforestation, following examples from the agriculture and food sectors (43). Such initiatives would increase the traceability and transparency of mining products and could thereby help slow down mining-induced deforestation as part of national and global forest conservation efforts.

## Materials and Methods

The bases for quantifying deforestation are 3,446 industrial mining extents (polygons with mining activities) for coal, metal ores, and industrial minerals identified by visual interpretation of the most recently available satellite images in an earlier study (31, 44) (*SI Appendix*, Fig. S1). These polygons were drawn around the geographical coordinates of mines reported in the Standard and Poor’s Metals and Mining database (45). All mines considered in our study were active in the time period from 2000 to 2019 (i.e., reported production in any of the years). We intersect these polygons with areas of tropical forest loss over the period from 2000 to 2019 using the 1-arcsec resolution Global Forest Change dataset (23) for all terrestrial tropical biomes defined in the Ecoregions 2017 dataset (46), including wet and dry tropical forests. Indirect forest loss is modeled as a function of proximity to mines, following earlier studies (9). The regression model controls for a range of other factors that have been identified as key factors in tropical deforestation (47, 48) (*SI Appendix*, Fig. S3). These include physical characteristics (elevation, slope, soil type), population density, and proximity to protected areas and to agriculture as well as proximity to roads and navigable waterways (*SI Appendix*, Table S1). Furthermore, the scale of mining was addressed by taking the spatial extent of mine features into consideration. Causal effects are estimated using linear and logistic regressions per country. Data sources and availabilities as well as the statistical framework are described in detail in *SI Appendix*.

## Supplementary Material

Supplementary File

## Data Availability

The data sets have been deposited in a publicly accessible database, PANGAEA https://doi.pangaea.de/10.1594/PANGAEA.928573 (49). All code to process the geospatial data and perform the statistical assessment is available on GitHub from https://github.com/fineprint-global/mining_deforestation-data-preparation (50) and https://github.com/fineprint-global/mining_deforestation-stat (51).
