# Supplementary material for: A pantropical assessment of deforestation caused by industrial mining
Source: Proc Natl Acad Sci U S A. 2022 Sep 12;119(38):e2118273119. doi: 10.1073/pnas.2118273119 (PMC9499560; doi:10.1073/pnas.2118273119)
Supplement: Supplementary File [file pnas.2118273119.sapp.pdf]

PNAS
www.pnas.org

Supplementary Information for

A pan-tropical assessment of deforestation caused by industrial mining

S. Giljum, V. Maus, N. Kuschnig, S. Luckeneder, M. Tost, L. Sonter, A. Bebbington

Corresponding Authors: Anthony Bebbington, Stefan Giljum
E-mail: abebbington@clarku.edu, stefan.giljum@wu.ac.at

This PDF file includes:

Supplementary text
Figs. S1 to S4 (not allowed for Brief Reports)
Tables S1 to S8 (not allowed for Brief Reports)
SI References

Supporting Information Text

Geospatial data

Study area. Deforestation is observed across all types of ecosystems, but it is a particular issue in the tropics. Tropical regions are unique in their high coverage with primary forests, their richness in species and their great significance for the stability of the global climate system, while at the same time facing alarming deforestation rates (1, 2). Therefore, we limit the scope of this study to terrestrial tropical biomes according to the Ecoregions data set classification (3, 4).

The three main criteria applied for the selection of the set of countries were (a) the presence of a sufficient number of industrial mining sites as reported in the SNL Metals and Mining Database (5) and therefore included in the set of mining polygons, (b) the location of the polygons in tropical forest areas, and (c) the importance of a country in terms of absolute forest loss observed since 2000. For many tropical countries, the data set used for mining polygons (6) contains only a very small number of mines. For example, in countries such as Bangladesh, Costa Rica or Madagascar, only one single mine is represented. The applied statistical methods exploit variation across observations; in order to avoid distorted results we excluded countries with fewer than three distinct mining sites. In other important mining countries, such as Peru and Bolivia, the industrial mines reported in the SNL database are located in biomes other than tropical forests, whereas deforestation occurs due to artisanal and small-scale mining (ASM) (7, 8). Due to this lack of industrial mines in tropical forests, these countries were also excluded. The final set of 26 countries represent 76.7 per cent of total tropical forest loss observed in the period of 2000 to 2019. The 26 countries include 3,446 polygons and a total mining area of 11,467 km^2 with a forest cover in 2000 of 7,019 km^2 (see Supplementary Table 2).

Spatial grid. We performed our analysis at 30 arcsec spatial resolution (approximately 1 by 1 km at the equator). A 30 arcsec grid was selected because it is the highest spatial resolution to which all required variables could be resampled without downscaling. For that we assembled a global grid data set, including the target variables of our model (forest cover loss and distance to mines) as well as the set of control variables.

Distance to mines and mining area. Proximity to mines is our explanatory variable for which we estimated the effect on forest cover loss. To produce this layer we used a global-scale data set of mining polygons published in previous work (9). The polygons represent mining extents for the extraction of coal, metal ores and industrial minerals. These mining areas were delineated by visual interpretation of satellite images around the year 2018 using several sources, including Google Earth, Microsoft Bing Imagery and Sentinel-2 cloudless (10). The mining areas are defined as all land used to extract raw materials from the Earth, including any step in extraction and processing by the mining sector. This definition includes ground features, such as open cuts, tailings dams, waste rock dumps, water ponds, processing plants, power plants, water storage dams, and other infrastructure that could be clearly linked to mining using satellite images. The overall accuracy of data is 88.4 % with commission error under 2.5 % and omission error around 21.2 %). Most mapping errors occurred within a 50 m buffer from polygons borders, indicating imprecision in polygons geometries rather than thematic mapping errors (9). The scale of the geometry errors (meters) do not affect the present study as our scale of analysis is the order of kilometres. We aggregated the area of the polygons to a 30 arcsec grid. The mining area for each grid cell was calculated by the intersection between the grid cells and the polygons. From this layer we derived the 'distance to mine' variable, which is the distance from the centroid of each grid cell to the centroid of the closest grid cell with a mining area greater than zero.

Note that we did not calculate direct deforestation by type of commodity. In a majority of cases, metal ores are extracted as 'polymetallic ores', i.e. one body of crude ore contains a number of different metal elements (11, 12). Estimating the mining area and related deforestation by single metal elements would have required developing an approach to cluster mining polygons with SNL coordinates and allocating deforestation areas to different commodities based on production volumes or prices. This was beyond the scope of this paper.

Area of forest cover and accumulated loss. Area of forest cover loss is our dependent variable. We used the GFC data set (13) to calculate the area of forest loss accumulated from 2000 to 2019. The GFC data set provides information on tree cover and tree loss at 1 arcsec resolution (approximately 30 by 30 m at the equator) on a global scale. For some regions, tree cover also includes plantation forests (13), however, we assume this to play a minor role in the context of mining-induced deforestation and therefore use the term forest loss instead of tree loss in our study. We aggregated the forest cover loss from 1 arcsec to our 30 arcsec grid cells by summing the area of forest loss pixels weighted by their surface intersection with the 30 arcsec cells (14). We calculated the direct forest cover loss due to mining by intersecting the forest cover loss data at 1 arcsec with the mining polygons (9) then aggregating these results to the 30 arcsec grid cells, which is the scale of our analysis. The area of forest cover in the year 2000 for each grid cell within and outside mining areas was calculated using the same approach as the forest cover loss.

Additional control variables. In addition to our explanatory variable of interest (proximity to mine), mining area and the initial forest extent (forest cover in 2000) we controlled for eleven variables known to determine the spatial distribution of tropical deforestation. Various spatial data layers from different sources were integrated to the 30 arcsec grid.

Population density was available at 30 arcsec resolution (15) and did not require further processing. Proximity to major transport routes as well as rivers were derived from Open Street Map (OSM). We intersected the OSM data with our 30 arcsec grid to produce layers with distances to highways and to navigable waterways. The distance was calculated from the centroid of each grid cell to the centroid of the closest grid cell with the respective type of infrastructure. A distance to protected areas

was also calculated. The protected areas vector data from UNEP-WCMC (16) was converted into a 30 arcsec binary grid, from which we calculated the distances from each cell to the closest protected cell. We did not distinguish between different types of protected areas.

Other land-cover variables for the year 2000 were resampled from the 300 m Climate Change Initiative Land Cover maps (17) to our grid using the major class present in each cell. From this layer we also calculated the distance to agriculture as the distances from the centroid of each cell to the closest agriculture cell. Note that we were not able to include a variable on "distance to logging" due to a lack of data across all investigated countries.

Finally, biophysical characteristics, including slope and elevation available at 30 arcsec resolution (18, 19) were integrated to our data set without further processing. Soil classes from SoilGrids™ (20–22) were resampled from 250 meters resolution to 30 arcsec by major class present in each grid cell.

Data availability. All data sets used in our analysis are openly available. Slope and Elevation (18, 19) are available at 30 arcsec resolution from https://doi.pangaea.de/10.1594/PANGAEA.867115 and population density (15) from https://sedac.ciesin.columbia.edu/data/set/gpw-v4-population-density-rev11. Forest cover and forest loss from GFC (13) are available from https://earthenginepartners.appspot.com/science-2013-global-forest/download_v1.7.html at 1 arcsec spatial resolution. Our resampled forest cover and forest loss area at 30 arcsec (6) is available from PANGAEA at https://doi.pangaea.de/10.1594/PANGAEA.928573. This data repository also contains the mining area, distance to mines, and the Ecoregions (3, 4) at 30 arcsec. The world protected areas (16) used to calculate distance to protected area is available from https://www.protectedplanet.net/en/thematic-areas/wdpa?tab=WDPA. Major transport routes and rivers are available from https://wiki.openstreetmap.org/wiki/Downloading_data. Land-cover for the year 2000 (17) is available from https://maps.elie.ucl.ac.be/CCI/viewer/ and soil classes from SoilGrids™(20–22) at https://www.isric.org/explore/soilgrids.

All code to process the geospatial data and perform the statistical assessment is available on GitHub. The data preparation repository can be found at https://github.com/fineprint-global/mining_deforestation-data-preparation and the repository containing the statistical modelling can be found at https://github.com/fineprint-global/mining_deforestation-stat.

Statistical framework

We investigated indirect effects of mining on forest loss via regression analysis. Our grid cell data were pruned using matching, in order to limit model dependence and emulate a fully-blocked experimental setting (23). The model specification was aimed at controlling for the confounding effects discussed above, in order to capture causal effects of mining on forest loss along our hypothesised pathway (24).

Identifying the drivers of deforestation. To integrate all relevant effects into our statistical model, we first developed a conceptual framework of potential causal pathways leading to tropical forest loss (Supplementary Figure 2). Drivers of deforestation were derived from (meta) studies on deforestation that identified the key determinants of tropical forest loss (25–28). Note that our framework also considers factors that lower deforestation, such as steep slopes, high elevations and protected areas.

For our statistical model, we selected 'proximity to mine' as the treatment variable, 'forest cover loss' as the dependent variable and a set of additional control variables, all available on a global scale and at a resolution of 1 by 1 km (30 arc seconds, see Supplementary Table 1 for a summary). Indirect forest loss driven by mineral extraction, modelled through the proximity of each grid cell to the nearest mine, is of our central interest. In order to specify causal effects of mining on deforestation, while taking into account a range of other factors, we selected eleven control variables. As production data per mining polygon was not available, the direct land use of mining, i.e. the areal extent of all polygons within one grid cell, was used as a proxy variable for mining intensity, assuming that deforestation would not only depend on the distance to a mine, but also on the size of the mining project. Areal extent served as a suitable proxy, because production and area of mines were shown to strongly correlate (29). We included three variables controlling for the distribution in the initial year 2000: forest cover to control for forest distribution, land use to reflect the initial distribution between different types of land (cropland, forests, etc.) and population density as a proxy for urban development and economic activity. We controlled for agricultural activity by including a variable on the distance of each grid cell to the nearest agricultural area. Further, we considered the effects of proximity to access infrastructure and transport modes by including a variable of proximity to waterways (i.e. rivers) and another on proximity to major roads such as highways. We assume major roads to be determined exogenously from mining sites, while we regard smaller types of roads as one of the mediators between mining activities and deforestation. We also included a control variable of proximity to protected areas as an environmental policy factor. Finally, we considered three variables of biophysical characteristics of each grid cell: soil type, slope and elevation.

Coarsened exact matching. For our analysis, we divided the global data set into subsets of single countries instead of pooling them. Countries were thus addressed individually and all relevant factors may have country-specific impacts. This is important because dynamics may differ considerably across countries and national circumstances may impact the effects of, e.g., agriculture or protected areas on forest loss. Within a given country, only a subset of locations was relevant to our analysis of forest loss and mining with imbalances across covariates. To limit the degree of imbalance and model dependence, we used a coarsened exact matching (CEM) approach (23, 30). CEM emulates a fully blocked randomised experiment instead of the more inefficient and imbalance-inducing fully randomised experiment applied in the propensity score matching approach (31) that was also used in previous mining-related studies (32). In addition, CEM does not rely on a dimension reduction and explicitly finds matches across all considered covariates.

Taking Ghana as an example, there were 280,193 observations of 30 arcsec grid cells for the entire country. A closer look reveals considerable natural and structural differences within the country. The northern half of the country's surface is not covered by forest, and geological conditions are of little use to mining. We seeked to balance observations in mining areas with ones outside, with a cutoff at 50 kilometres (i.e. all observations from the control group were located at least 50 km from mines and all treated were within this threshold; no further matching limitations were specified). Decision on this cut-off was taken based on earlier studies finding that indirect impacts of mining on deforestation in the Brazilian Amazon occur within a 50 km range around mines (32). In accordance with the literature (33), we took a tentative approach – matching on the most important variables and pruning cautiously. For the exemplary case of Ghana, our approach yielded 36,982 observations i.e. approximately 13 percent of the unadjusted sample. The procedure was successful in balancing observations close and far from mines with regard to all considered characteristics. More detail on matching performance is provided in Supplementary Figure 3.

Linear regression. With the resulting matched data, we considered a linear regression model of the form

$$\mathbf{y}_i = \alpha_i + \mathbf{x}_i\delta_i + \mathbf{Z}_i\theta_i + \mathbf{e}_i. \tag{1}$$

The subscript $i \in [1, I]$ indicates country subsets, which include N_i observations. The dependent variable $\mathbf{y}_i \in \mathbb{R}^{N_i}$ is log-transformed total forest loss in square meters. The treatment variable $\mathbf{x}_i \in \mathbb{R}^{N_i}$ contains log-transformed distances to the nearest mine. The matrix $\mathbf{Z}_i \in \mathbb{R}^{N_i \times K}$ contains a set of K control variables (see Section on spatial grid above) and functions thereof. Lastly, $\mathbf{e}_i$ is an error term with an iid Gaussian distribution with mean zero and constant variance σ^2.

The linear regression model has some major advantages – it allows us to connect the theoretical setup to the data and yields interpretable outputs (34). One required assumption is linearity in parameters. However, it should be noted that this did not prevent us from capturing a variety of non-linear effects. Most importantly, forest loss and mine distance are not related linearly (32) – i.e. we do not expect the same effects from increasing the distance from one to two kilometres and from 99 to 100 kilometres. Instead, we considered the relation in relative terms, i.e. as an elasticity. By log-transforming both variables the coefficient δ_i gives the percent change in forest loss from a one percent change in distance, with all other values equal. We applied the same log-transformation to distance variables, given that impacts fade out with increased distance. For these variables, we additionally allowed for discontinuous effects by considering thresholds at 5, 10, 25, and 50 kilometres. In order to check for robustness, we tested our results against a number of alternative specifications (Supplementary Tables 5 and 6).

Another important limitation of the linear model is due to the nature of our dependent variable. There are lower and upper bounds to forest loss, with a considerable number of observations at the lower bound of zero. The linear model does not account for these cutoffs and other approaches may be warranted (34). We considered this and alternatively estimated non-linear variants – (1) a Logit model with logistic link function to estimate the share of forest loss per area, and (2) a Tobit model to address the censored dependent variable. Both variations address issues with the limited dependent variable appropriately and yield more efficient estimates. This improved fit came at the cost of interpretability. Coefficients cannot be interpreted as partial derivatives anymore, but must be seen in context of the non-linear link transformation. To avoid this source of confusion and misinterpretation, we considered these models as robustness checks for the simpler linear model. Results from both approaches largely mirror the linear model, supporting our modelling choice. Estimates from both alternative models are available in Supplementary Table 7.

Hypothetical expansion scenario. For illustrating the coefficient estimates, we computed the indirect deforestation effect of an expansion of all mines in each country. We assumed a 100 m spread of mining polygons, i.e. we created a counterfactual data set, for which we reduced the distances to a mine by 100 m and computed Δ_{ri}, the relative change in distance to a mine of such as scenario for each grid cell $r \in [0, R]$ in country i. Observations closer than 100 m to a mining polygon before expansion were excluded, as they needed to be considered as direct deforestation. The indirect deforestation effect was then calculated as the accumulated difference between reported forest loss, L_{ri}, and the estimated forest loss including indirect deforestation due to mine expansion, L^*_{ri}. The estimates for L^*_{ri} were computed in accordance with the model in Equation 1 as

$$L^*_{ri} = e^{\Delta_{ri}\delta_i + y_{ri}} \tag{2}$$

with Δ_{ri} denoting the relative change in distance to mine, δ_i being the country-specific coefficient and y_{ri} the log-transformed total forest loss in square meters.

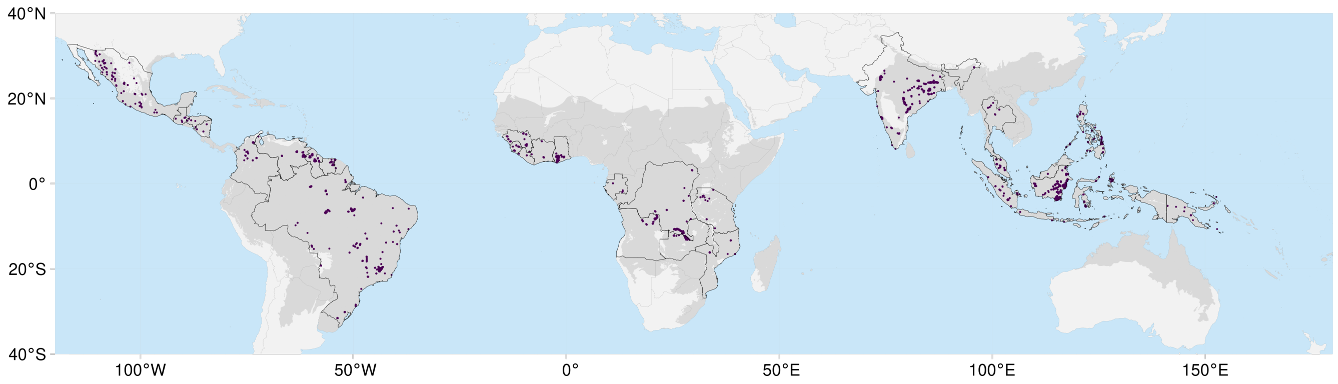

Fig. S1. The 3,446 mining polygons (9, 35) in the 26 countries with tropical forests considered in this study. The shaded areas represent the extent of tropical biomes. Dark borders surround the countries included in this study and the points indicate the centroids of mines.

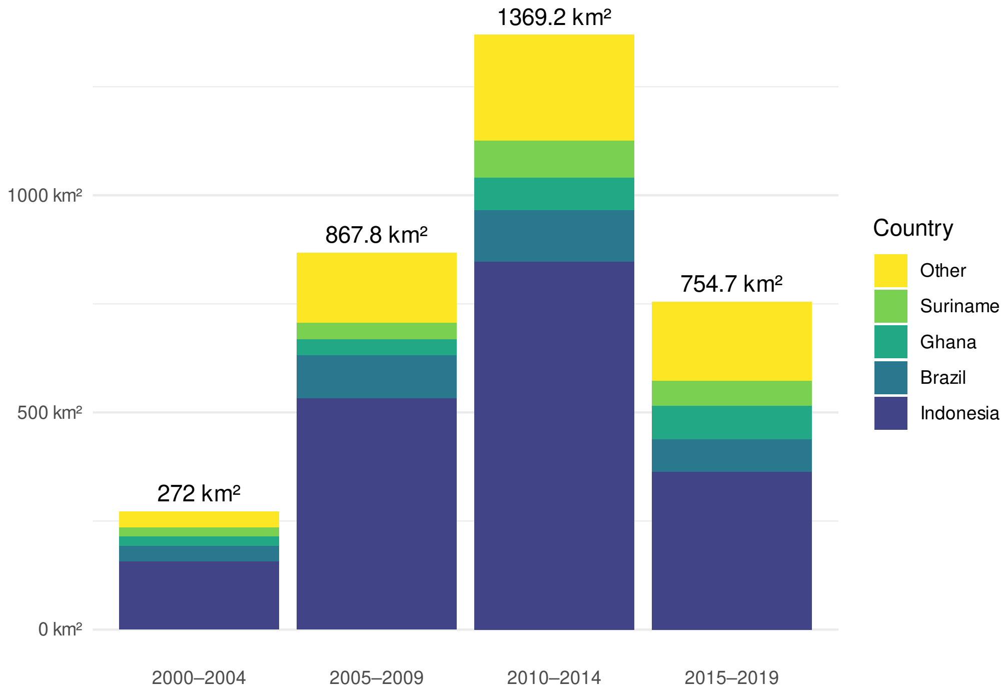

Fig. S2. Direct deforestation within mining areas per 5-year time period. Top-4 countries (Indonesia, Brazil, Ghana and Suriname) plus 22 other countries aggregated.

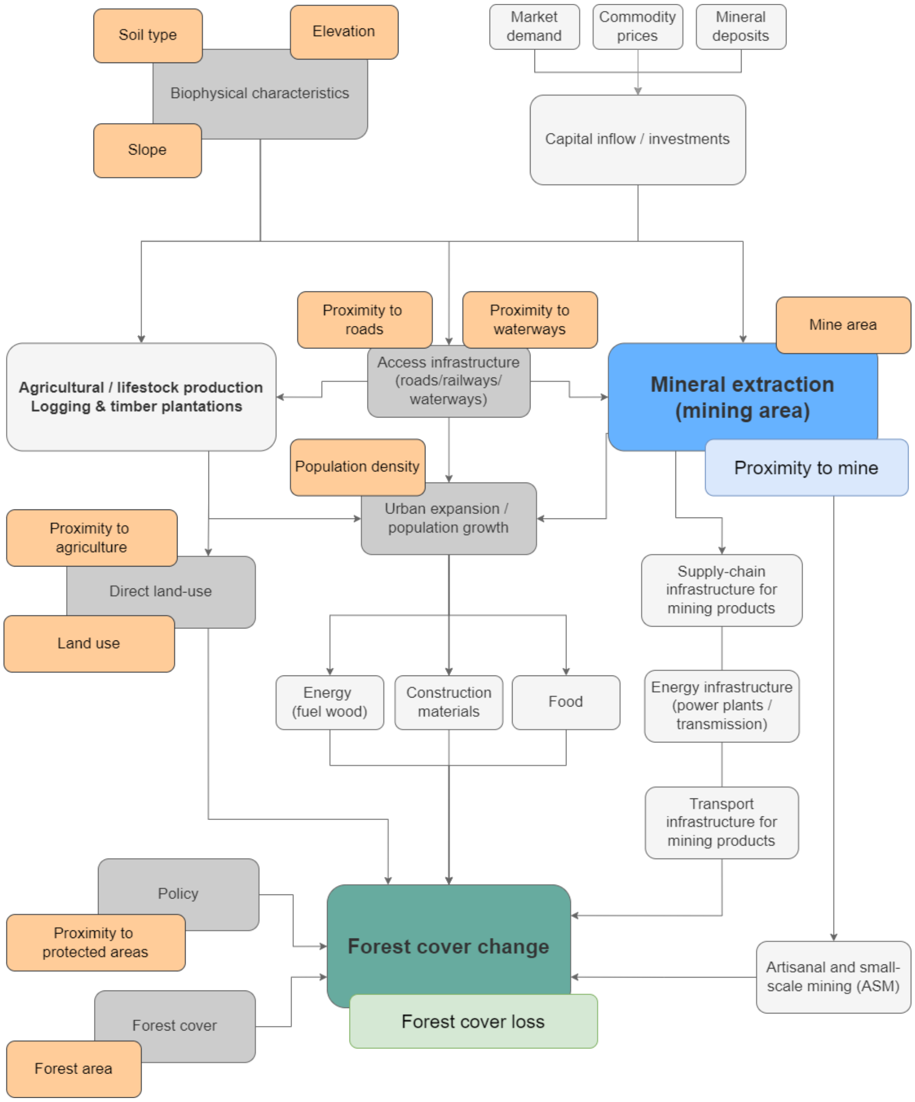

Fig. S3. Identification of deforestation drivers and corresponding variables. The flow diagram indicates potential pathways leading to forest cover loss and the variables considered in this study. Dark grey boxes illustrate those drivers that could be covered with control variables, marked with orange boxes. Light grey boxes illustrate drivers that could not be considered in the regression model.

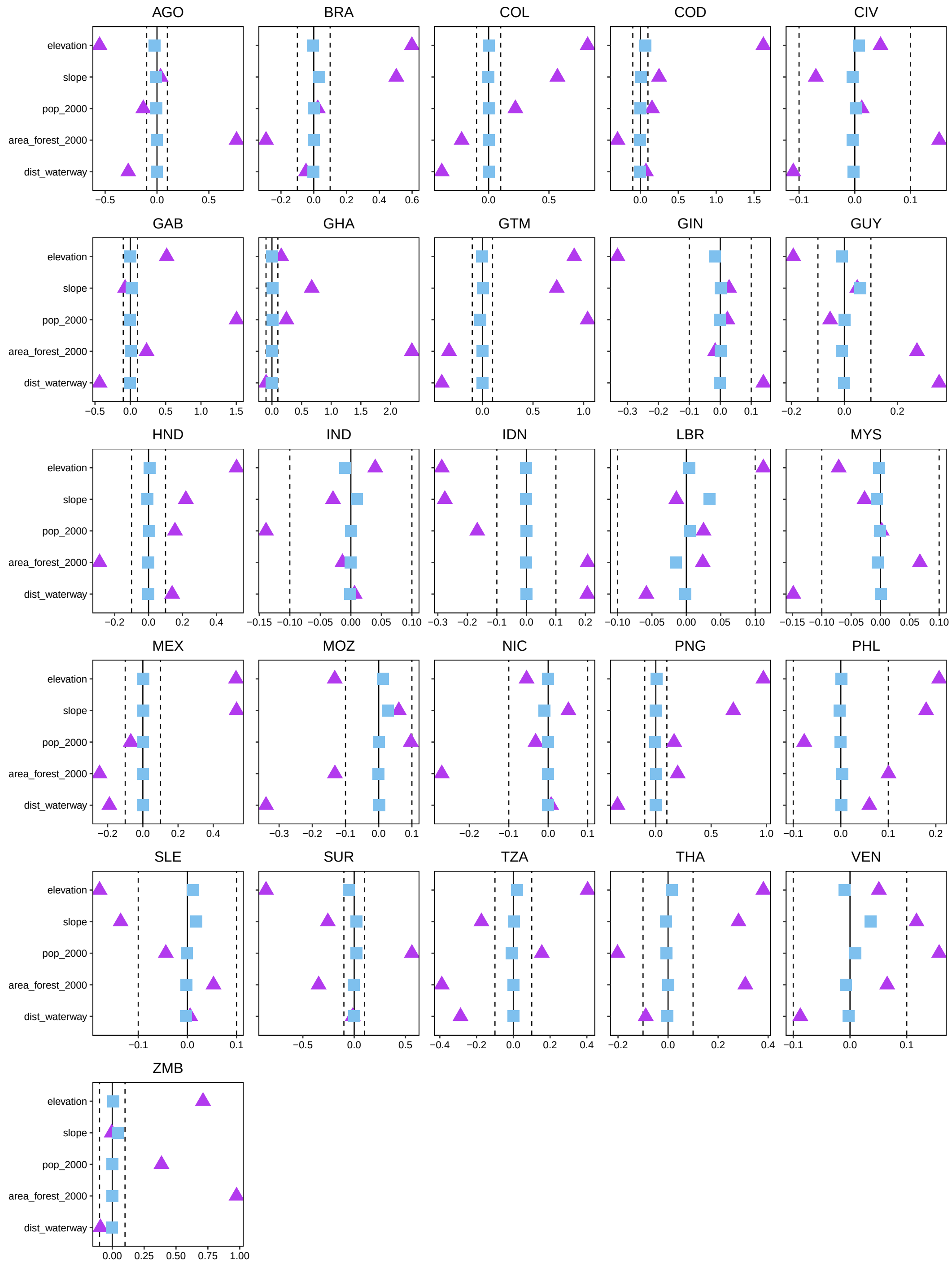

Fig. S4. Standardised mean differences of the matching variables before (triangle) and after (square) matching. Dashed lines indicate an upper limit threshold of 0.1. Categorical variables (ecosystems, ESA CCI landcover and soil types) were excluded for reasons of space. Plots were created in R using the cobalt package (36). Countries are indicated by their respective ISO 3166-1 alpha-3 code.

Table S1. Set of variables used in the statistical analysis

Variable	Specification	Source
**Dependent**		
Forest cover loss	2000-2019 accumulated deforestation	Calculation based on Hansen et al. (2013) (13)
**Treatment**		
Proximity to mine	Derived from mine polygons	Calculation based on Maus et al. (2020) (35)
**Controls**		
Mining area	Mining intensity	Calculation based on Maus et al. (2020) (35)
Forest area in 2000	Forest convergence	Hansen et al. (2013)(13)
Land use in 2000	Croplands, forest, shrubbery, etc.	ESA Climate Change Initiative(17)
Population density in 2000	Human activity	NASA (2018)(15)
Proximity to agriculture	Agricultural activity	ESA Climate Change Initiative(17)
Proximity to waterways	Rivers	Open Street Map (planet.openstreetmap.org)
Proximity to roads	Highway and trunk classifications	Open Street Map (planet.openstreetmap.org)
Proximity to protected areas	Institutional factor	Calculation based on UNEP-WCMC (2020) (16)
Slope	Biophysical characteristics	Amatulli et al. (2018)(18)
Elevation	Biophysical characteristics	Amatulli et al. (2018)(18)
Soil type	Agricultural suitability	SoilGrids™(22)

Table S2. Mining area in km^2, Number of polygons, Forest area in 2000 in km^2, Accumulated deforestation 2000-2019 in km^2 and Percentage share of deforestation in total forest area within mining polygons. Countries are indicated by their respective ISO 3166-1 alpha-3 code.

	Mining area	Polygons	Forest area, 2000	Deforestation, 2000-2019	Percentage
AGO	179.22	75	71.38	31.35	43.92
BRA	1485.08	446	745.10	327.14	43.91
CIV	37.21	9	16.61	11.09	66.75
COD	367.38	139	155.91	98.75	63.34
COL	328.40	52	221.71	68.32	30.81
GAB	46.07	7	31.12	1.55	4.97
GHA	657.50	273	617.01	212.52	34.44
GIN	146.56	67	4.57	2.92	63.85
GTM	9.72	13	2.74	1.05	38.42
GUY	237.14	132	431.19	80.87	18.76
HND	7.15	7	1.45	0.65	44.60
IDN	3576.69	883	2841.45	1900.98	66.90
IND	1578.31	690	85.46	13.47	15.76
LBR	11.65	18	9.77	5.67	58.03
MEX	235.28	153	48.59	16.81	34.59
MOZ	75.57	21	3.47	2.10	60.64
MYS	63.38	53	39.72	27.99	70.48
NIC	9.48	9	3.71	0.79	21.32
PHL	217.91	123	84.55	29.59	34.99
PNG	51.98	14	32.31	10.20	31.57
SLE	39.06	50	32.93	14.11	42.84
SUR	791.42	57	722.29	202.61	28.05
THA	130.72	13	13.83	3.11	22.46
TZA	98.57	19	16.50	7.54	45.68
VEN	652.75	48	553.88	97.55	17.61
ZMB	432.56	75	231.68	94.92	40.97
TOTAL	11466.73	3446	7018.90	3263.62	46.50

Table S3. Direct (on-site) deforestation per time period and country, in km^2. Countries are indicated by their respective ISO 3166-1 alpha-3 code.

	2000-2004	2005-2009	2010-2014	2015-2019	TOTAL
AGO	3.75	11.42	8.55	7.62	31.35
BRA	35.29	98.72	118.37	74.76	327.14
CIV	0.18	1.41	3.77	5.72	11.09
COD	2.47	20.85	37.83	37.60	98.75
COL	6.15	16.13	27.57	18.47	68.32
GAB	0.23	0.33	0.54	0.45	1.55
GHA	22.70	37.24	75.51	77.07	212.52
GIN	0.61	1.30	0.55	0.46	2.92
GTM	0.13	0.44	0.35	0.14	1.05
GUY	3.77	19.32	31.81	25.97	80.87
HND	0.16	0.23	0.20	0.04	0.65
IDN	157.52	532.95	847.28	363.23	1900.98
IND	1.06	6.71	3.60	2.09	13.47
LBR	0.13	0.23	3.32	2.00	5.67
MEX	0.69	5.69	6.60	3.83	16.81
MOZ	0.11	1.00	0.89	0.10	2.10
MYS	2.23	8.09	13.83	3.85	27.99
NIC	0.12	0.25	0.39	0.04	0.79
PHL	0.93	7.52	14.58	6.54	29.59
PNG	1.52	5.69	2.55	0.44	10.20
SLE	0.32	3.51	6.63	3.64	14.11
SUR	20.87	37.82	85.33	58.60	202.61
THA	0.47	1.42	0.86	0.36	3.11
TZA	1.69	2.60	1.05	2.20	7.54
VEN	6.30	23.49	30.80	36.96	97.55
ZMB	2.54	23.44	46.46	22.48	94.92
TOTAL	271.96	867.79	1369.20	754.66	3263.62

Table S4. Main model OLS estimates for all control variables except interaction terms and categorical landcover, soilgrid and biome variables. Standard errors in parentheses. aforest = area of forest in 2000, droad = distance to road, dwater = distance to waterway, dcrop = distance to cropland, dmine = distance to mine, dpa = distance to protected area, elev = elevation, amine = areal extent of mining, pop = population density in 2000, Obs. = no. of observations. Countries are indicated by their respective ISO 3166-1 alpha-3 code.

Country	aforest	droad	dwater	dcrop	dmine	dpa	elev	amine	pop	slope	Obs.	Share treated	R^2
AGO	0.300	-0.036	-0.154	-0.454	-0.208	0.068	0.0015	-0.032	-0.425	0.029	67993	0.48	0.08
	0.030	0.142	0.012	0.068	0.027	0.017	0.0003	0.001	0.701	0.107			
BRA	0.527	0.018	-0.122	-0.947	-0.306	0.144	0.0003	-0.003	-2.264	0.104	2273696	0.13	0.34
	0.001	0.004	0.007	0.006	0.004	0.001	0.0000	0.000	0.053	0.003			
CIV	0.940	-0.133	0.039	-0.038	0.086	0.030	-0.0010	-0.001	-0.444	0.145	72565	0.29	0.98
	0.004	0.017	0.003	0.012	0.004	0.004	0.0001	0.000	0.049	0.014			
COD	0.266	-1.092	-0.238	-1.603	-0.141	0.049	-0.0008	0.005	-6.162	-0.201	280658	0.26	0.18
	0.023	0.033	0.038	0.019	0.013	0.003	0.0001	0.000	0.163	0.017			
COL	0.732	0.196	-0.097	-0.677	-0.321	0.174	-0.0016	-0.006	0.812	-0.025	38125	0.45	0.34
	0.079	0.067	0.106	0.038	0.021	0.009	0.0001	0.000	0.342	0.005			
	0.743	0.077	0.125	0.115	0.063	0.069	0.0002	0.034	3.387	0.020			
GAB	-9.919	-2.520	-0.109	-0.752	-1.965	0.120	-0.0119	-0.006	40.395	-0.416	6588	0.44	0.13
	8.036	0.347	0.093	0.163	0.122	0.043	0.0015	0.004	54.455	0.193			
GHA	0.559	-0.014	0.033	-0.530	-0.037	0.070	-0.0019	0.002	-0.044	-0.103	36982	0.59	0.95
	0.010	0.028	0.005	0.020	0.011	0.006	0.0002	0.000	0.068	0.017			
GIN	0.622	-0.553	0.068	-0.335	0.371	0.046	0.0004	0.000	-2.038	0.187	72633	0.44	0.81
	0.006	0.022	0.011	0.023	0.015	0.004	0.0001	0.001	0.087	0.021			
GTM	0.238	1.019	0.055	0.021	-0.073	0.161	-0.0004	0.025	-0.482	0.025	8604	0.40	0.29
	0.906	0.167	0.018	0.067	0.079	0.012	0.0001	0.024	2.588	0.010			
GUY	-1.388	-0.313	-0.133	-0.684	-0.551	0.060	-0.0020	-0.000	69.242	-0.059	163572	0.39	0.11
	0.147	0.025	0.013	0.031	0.013	0.004	0.0001	0.000	10.626	0.008			
HND	2.021	-0.463	0.058	0.060	-0.178	0.071	-0.0007	0.079	-0.281	-0.001	3325	0.48	0.30
	0.303	0.152	0.025	0.084	0.047	0.017	0.0003	0.021	1.000	0.024			
IDN	0.550	-0.365	-0.132	-0.790	-0.227	0.119	-0.0054	-0.001	-2.596	-0.139	621704	0.38	0.46
	0.015	0.010	0.008	0.010	0.006	0.002	0.0000	0.000	0.073	0.002			
IND	0.590	-0.074	-0.002	-0.146	0.015	0.013	-0.0003	-0.001	-0.136	-0.099	846387	0.30	0.37
	0.003	0.005	0.005	0.011	0.002	0.001	0.0000	0.000	0.007	0.003			
	0.072	0.405	0.048	1.408	0.202	0.088	0.0005	0.020	0.761	0.271			
LBR	-63.929	0.223	0.054	-2.332	-0.279	0.200	-0.0038	0.027	-230.779	-0.242	41739	0.34	0.43
	4.793	0.059	0.045	0.037	0.022	0.007	0.0004	0.006	19.236	0.044			
MEX	0.154	0.002	0.037	0.069	-0.082	0.020	-0.0012	0.002	0.265	0.060	152740	0.46	0.37
	0.002	0.012	0.007	0.019	0.012	0.003	0.0000	0.001	0.034	0.004			
MOZ	0.329	-1.023	-0.229	-0.362	0.822	0.023	0.0018	0.015	-4.243	-0.196	48977	0.32	0.56
	0.010	0.061	0.015	0.038	0.022	0.005	0.0002	0.001	0.260	0.044			
MYS	-0.297	-0.056	0.097	-0.532	-0.036	0.360	-0.0078	0.026	-5.266	-0.057	79174	0.44	0.34
	0.267	0.026	0.006	0.029	0.019	0.008	0.0002	0.002	0.682	0.006			
NIC	0.785	-0.735	-0.034	-0.599	-0.493	-0.057	-0.0073	0.008	-4.388	-0.223	20153	0.35	0.62
	0.074	0.134	0.011	0.068	0.044	0.016	0.0005	0.024	0.474	0.021			
PHL	0.751	-0.146	-0.029	-0.202	-0.253	0.053	-0.0026	-0.023	-0.667	-0.021	57652	0.39	0.34
	0.060	0.033	0.021	0.147	0.018	0.008	0.0001	0.001	0.134	0.005			
PNG	-0.185	1.299	-0.114	-1.059	-0.241	-0.040	-0.0028	-0.012	39.370	0.047	63933	0.34	0.21
	0.185	0.103	0.010	0.044	0.025	0.011	0.0001	0.003	3.012	0.005			
SLE	1.711	0.122	0.026	-1.171	-0.079	0.125	0.0010	0.007	2.077	-0.039	17961	0.60	0.50
	0.182	0.048	0.006	0.048	0.022	0.015	0.0002	0.001	0.631	0.015			
SUR	-13.624	0.404	-0.039	-0.520	-0.874	-0.026	0.0009	0.000	-37.898	0.283	105970	0.27	0.10
	0.561	0.095	0.007	0.041	0.020	0.004	0.0004	0.000	16.158	0.015			
THA	0.713	0.302	-0.039	0.192	0.349	0.181	-0.0012	0.007	0.430	-0.074	77423	0.25	0.53
	0.024	0.072	0.096	0.291	0.015	0.007	0.0001	0.001	0.154	0.014			
TZA	0.499	0.043	0.081	-1.337	0.090	0.023	-0.0005	-0.028	-0.137	-0.355	69522	0.42	0.67
	0.010	0.030	0.005	0.105	0.014	0.007	0.0001	0.001	0.072	0.089			
VEN	0.330	-0.081	-0.119	-0.898	-0.168	-0.062	-0.0006	-0.002	-0.279	0.043	187993	0.15	0.17
	0.007	0.016	0.015	0.025	0.019	0.004	0.0001	0.000	0.194	0.005			
	0.121	0.085	0.016	0.054	0.036	0.020	0.0002	0.001	0.221	0.012			
ZMB	-0.019	-0.550	-0.404	-0.939	-1.373	0.196	0.0043	-0.019	-4.582	2.067	96802	0.28	0.29
	0.090	0.044	0.020	0.036	0.023	0.091	0.0003	0.000	0.581	0.254			

Table S5. Indirect deforestation effects induced by mining in 29 tropical countries. Distance to mine (DM) OLS estimates in a model allowing for intersection terms at 5 km and 25 km distance from mine. Standard errors in parentheses; Obs. = no. of observations. Countries are indicated by their respective ISO 3166-1 alpha-3 code.

Country	DM	DM × 5 km	DM × 25 km	Obs.	Share treated	R^2
AGO	0.059	0.399	0.047	67993	0.48	0.08
	(0.036)	(0.028)	(0.007)			
BRA	-0.34	-0.208	0	2273696	0.13	0.34
	(0.004)	(0.006)	(0.002)			
CIV	0.049	0.019	-0.016	72565	0.29	0.98
	(0.006)	(0.005)	(0.001)			
COD	-0.069	0.067	0.047	280658	0.26	0.18
	(0.015)	(0.021)	(0.005)			
COL	-0.196	0.001	0.067	38125	0.45	0.34
	(0.025)	(0.021)	(0.007)			
	(0.084)	(0.042)	(0.02)			
GAB	-2.346	-0.254	-0.15	6588	0.44	0.13
	(0.142)	(0.487)	(0.029)			
GHA	-0.074	0.034	-0.022	36982	0.59	0.95
	(0.017)	(0.007)	(0.003)			
GIN	0.543	0.137	0.046	72633	0.44	0.81
	(0.02)	(0.015)	(0.004)			
GTM	-0.161	-0.111	-0.022	8604	0.40	0.29
	(0.092)	(0.096)	(0.014)			
GUY	-0.619	0.124	-0.052	163572	0.39	0.11
	(0.02)	(0.01)	(0.004)			
HND	-0.206	-0.104	0.003	3325	0.48	0.30
	(0.071)	(0.044)	(0.013)			
IDN	-0.354	-0.077	-0.046	621704	0.38	0.47
	(0.008)	(0.006)	(0.002)			
IND	0.014	-0.002	0	846387	0.30	0.37
	(0.002)	(0.002)	(0.001)			
	(0.217)	(0.315)	(0.057)			
LBR	-0.355	0.085	-0.049	41739	0.34	0.44
	(0.032)	(0.019)	(0.007)			
MEX	-0.122	0.016	-0.015	152740	0.46	0.37
	(0.016)	(0.012)	(0.003)			
MOZ	0.868	0.393	-0.001	48977	0.32	0.56
	(0.027)	(0.039)	(0.008)			
MYS	-0.148	-0.099	-0.028	79174	0.44	0.34
	(0.026)	(0.021)	(0.005)			
NIC	-0.506	-0.227	0.002	20153	0.35	0.62
	(0.052)	(0.097)	(0.013)			
PHL	-0.308	-0.077	-0.009	57652	0.39	0.34
	(0.024)	(0.015)	(0.006)			
PNG	-0.4	-0.031	-0.084	63933	0.34	0.21
	(0.031)	(0.025)	(0.009)			
SLE	-0.121	-0.027	-0.006	17961	0.60	0.50
	(0.034)	(0.016)	(0.005)			
SUR	-0.261	0.271	0.151	105970	0.27	0.10
	(0.028)	(0.012)	(0.006)			
THA	0.289	0.057	-0.046	77423	0.25	0.53
	(0.018)	(0.018)	(0.006)			
TZA	0.396	0.048	0.119	69522	0.42	0.67
	(0.018)	(0.016)	(0.004)			
VEN	-0.2	0.091	-0.069	187993	0.15	0.17
	(0.021)	(0.014)	(0.007)			
	(0.044)	(0.042)	(0.011)			
ZMB	-1.42	-0.275	0.038	96802	0.28	0.29
	(0.028)	(0.019)	(0.007)			

Table S6. Alternative model specifications indicated by subscripts: Model 1 represents distance to mine (log) OLS estimates (δ) and goodness of fit (R^2), where the dependent variable was changed to 2000–2010 forest cover loss. Model 2 is a sparse specification with only distance to mine, mining area, elevation, slope, population density and forest area in 2000 (and interaction terms) as covariates. 3 is our main model without interaction terms, and model 4 the main model except distance to road information. Standard errors in parentheses. Countries are indicated by their respective ISO 3166-1 alpha-3 code.

Country	δ_1	R_1^2	δ_2	R_2^2	δ_3	R_3^2	δ_4	R_4^2
AGO	-0.142	0.06	-0.138	0.06	-0.202	0.07	-0.23	0.08
	(0.026)		(0.026)		(0.027)		(0.025)	
BRA	-0.228	0.27	-0.592	0.12	-0.299	0.32	-0.305	0.33
	(0.004)		(0.004)		(0.004)		(0.004)	
CIV	0.078	0.84	0.082	0.98	0.094	0.98	0.091	0.98
	(0.011)		(0.004)		(0.004)		(0.004)	
COD	0.053	0.16	-0.656	0.05	-0.386	0.16	-0.228	0.18
	(0.013)		(0.013)		(0.013)		(0.013)	
COL	-0.198	0.30	-0.285	0.27	-0.315	0.32	-0.29	0.33
	(0.023)		(0.021)		(0.02)		(0.02)	
	(0.062)		(0.06)		(0.06)		(0.059)	
GAB	-1.357	0.13	-1.256	0.07	-1.82	0.10	-1.37	0.12
	(0.127)		(0.092)		(0.108)		(0.107)	
GHA	-0.027	0.87	-0.094	0.93	-0.022	0.95	-0.025	0.95
	(0.015)		(0.011)		(0.01)		(0.01)	
GIN	0.238	0.59	0.345	0.79	0.363	0.81	0.381	0.81
	(0.019)		(0.015)		(0.015)		(0.015)	
GTM	0.075	0.21	0.274	0.18	0.217	0.27	0.071	0.28
	(0.101)		(0.07)		(0.068)		(0.072)	
GUY	-0.22	0.11	-0.683	0.06	-0.656	0.08	-0.667	0.09
	(0.01)		(0.01)		(0.012)		(0.011)	
HND	0.455	0.12	-0.186	0.24	-0.148	0.28	-0.16	0.30
	(0.08)		(0.044)		(0.045)		(0.046)	
IDN	-0.128	0.45	-0.297	0.37	-0.23	0.44	-0.241	0.46
	(0.006)		(0.006)		(0.006)		(0.006)	
IND	0.02	0.29	0.01	0.35	0.012	0.36	0.013	0.37
	(0.002)		(0.002)		(0.002)		(0.002)	
	(0.203)		(0.16)		(0.165)		(0.195)	
LBR	-0.136	0.41	-0.664	0.19	-0.403	0.42	-0.274	0.43
	(0.024)		(0.023)		(0.02)		(0.021)	
MEX	-0.062	0.29	-0.098	0.31	-0.06	0.35	-0.084	0.37
	(0.01)		(0.012)		(0.012)		(0.012)	
MOZ	0.928	0.42	1.028	0.49	0.938	0.53	0.907	0.55
	(0.025)		(0.022)		(0.022)		(0.021)	
MYS	0.026	0.32	-0.121	0.23	0.015	0.32	-0.036	0.33
	(0.023)		(0.02)		(0.019)		(0.019)	
NIC	0.042	0.48	-1.157	0.44	-1.095	0.47	-0.509	0.61
	(0.056)		(0.051)		(0.051)		(0.044)	
PHL	-0.241	0.24	-0.32	0.29	-0.25	0.33	-0.247	0.33
	(0.02)		(0.018)		(0.017)		(0.017)	
PNG	-0.13	0.22	-0.417	0.13	-0.221	0.20	-0.398	0.20
	(0.024)		(0.025)		(0.025)		(0.025)	
SLE	0.246	0.31	-0.112	0.30	-0.101	0.48	-0.074	0.50
	(0.046)		(0.023)		(0.02)		(0.02)	
SUR	-0.393	0.06	-0.956	0.07	-0.872	0.08	-0.943	0.09
	(0.014)		(0.017)		(0.019)		(0.017)	
THA	0.488	0.40	0.259	0.50	0.258	0.53	0.352	0.53
	(0.015)		(0.014)		(0.014)		(0.015)	
TZA	0.185	0.52	-0.091	0.62	0.071	0.66	0.09	0.67
	(0.014)		(0.015)		(0.014)		(0.014)	
VEN	-0.047	0.18	-0.695	0.09	-0.014	0.16	-0.195	0.17
	(0.015)		(0.012)		(0.016)		(0.015)	
	(0.038)		(0.036)		(0.034)		(0.034)	
ZMB	-0.884	0.25	-1.393	0.13	-1.39	0.27	-1.606	0.28
	(0.023)		(0.023)		(0.022)		(0.02)	

Table S7. Indirect deforestation effects induced by mining in 29 tropical countries. Distance to mine (log) OLS (δ_{lm}), logit (δ_{logit}) and tobit (δ_{tobit}) estimates. Standard errors in parentheses; Obs. = no. of observations. Logit model uses share of forest cover loss relative to total area as dependent variable. Countries are indicated by their respective ISO 3166-1 alpha-3 code.

Country	δ_{lm}	δ_{logit}	δ_{tobit}	Obs.	Share treated	R^2_{lm}
AGO	-0.208	-0.514	-0.215	67993	0.48	0.08
	(0.027)	(0.044)	(0.051)			
BRA	-0.306	-0.085	-0.571	2273696	0.13	0.34
	(0.004)	(0.003)	(0.007)			
CIV	0.086	0.156	0.131	72565	0.29	0.98
	(0.004)	(0.02)	(0.005)			
COD	-0.141	-0.007	-0.23	280658	0.26	0.18
	(0.013)	(0.013)	(0.018)			
COL	-0.321	-0.097	-0.391	38125	0.45	0.34
	(0.021)	(0.027)	(0.025)			
	(0.063)	(0.108)	(0.091)			
GAB	-1.965	-0.471	-2.484	6588	0.44	0.13
	(0.122)	(0.261)	(0.197)			
GHA	-0.037	-0.172	-0.054	36982	0.59	0.95
	(0.011)	(0.026)	(0.017)			
GIN	0.371	0.214	0.567	72633	0.44	0.81
	(0.015)	(0.046)	(0.028)			
GTM	-0.073	-0.245	-0.08	8604	0.40	0.29
	(0.079)	(0.101)	(0.099)			
GUY	-0.551	-0.332	-1.418	163572	0.39	0.11
	(0.013)	(0.038)	(0.038)			
HND	-0.178	-0.151	-0.18	3325	0.48	0.30
	(0.047)	(0.087)	(0.044)			
IDN	-0.227	-0.056	-0.284	621704	0.38	0.46
	(0.006)	(0.004)	(0.007)			
IND	0.015	0.207	0.22	846387	0.30	0.37
	(0.002)	(0.079)	(0.068)			
	(0.202)	(1.058)	(0.52)			
LBR	-0.279	-0.245	-0.279	41739	0.34	0.43
	(0.022)	(0.019)	(0.021)			
MEX	-0.082	0.02	-0.375	152740	0.46	0.37
	(0.012)	(0.067)	(0.058)			
MOZ	0.822	0.374	1.057	48977	0.32	0.56
	(0.022)	(0.035)	(0.036)			
MYS	-0.036	0.107	-0.045	79174	0.44	0.34
	(0.019)	(0.014)	(0.022)			
NIC	-0.493	-0.149	-0.556	20153	0.35	0.62
	(0.044)	(0.044)	(0.051)			
PHL	-0.253	-0.365	-0.252	57652	0.39	0.34
	(0.018)	(0.022)	(0.021)			
PNG	-0.241	-0.037	-0.396	63933	0.34	0.21
	(0.025)	(0.031)	(0.044)			
SLE	-0.079	-0.085	-0.088	17961	0.60	0.50
	(0.022)	(0.04)	(0.019)			
SUR	-0.874	-0.512	-2.098	105970	0.27	0.10
	(0.02)	(0.042)	(0.059)			
THA	0.349	0.22	0.852	77423	0.25	0.53
	(0.015)	(0.073)	(0.058)			
TZA	0.09	-0.372	0.016	69522	0.42	0.67
	(0.014)	(0.051)	(0.052)			
VEN	-0.168	-0.159	-0.596	187993	0.15	0.17
	(0.019)	(0.041)	(0.058)			
	(0.036)	(0.043)	(0.06)			
ZMB	-1.373	-0.502	-1.775	96802	0.28	0.29
	(0.023)	(0.021)	(0.031)			

Table S8. Estimated deforestation in a scenario where all mines expand their borders by 100 m; lower and upper bound of 95% confidence interval. Selection of Top-10 countries with largest effects.

	Estimated effect (in km^2)	
Country	95% lower	95% upper
Indonesia	194	215
Brazil	147	154
Zambia	56	60
Suriname	17	19
Colombia	14	18
DR Congo	13	18
Nicaragua	10	15
Liberia	9	12
Gabon	9	12
Philippines	7	9

References

1. Deborah Lawrence, Karen Vandecar, Effects of tropical deforestation on climate and agriculture. *Nat. Clim. Chang.* **5**, 27–36 (2015).
2. Frances Seymour, Nancy L. Harris, Reducing tropical deforestation. *Science* **365**, 756–757 (2019).
3. E Dinerstein, et al., An ecoregion-based approach to protecting half the terrestrial realm. *BioScience* **67**, 534–545 (2017).
4. Resolve, *Ecoregions. http://ecoregions2017.appspot.com.* (2017).
5. SNL, *Metals and Mining Database.* (S&P Global Market Intelligence, New York), (2020).
6. V Maus, N Kuschnig, S Luckeneder, S Giljum, A set of essential variables for modelling environmental impacts of global mining land use (*PANGAEA* https://doi.pangaea.de/10.1594/PANGAEA.928573) (2022).
7. J Caballero Espejo, et al., Deforestation and forest degradation due to gold mining in the Peruvian Amazon: A 34-year perspective. *Remote. Sens.* **10** (2018).
8. GP Asner, R Tupayachi, Accelerated losses of protected forests from gold mining in the Peruvian amazon. *Environ. Res. Lett.* **12**, 094004 (2016).
9. V Maus, et al., A global-scale data set of mining areas. *Sci. Data* **7**, 289 (2020).
10. EOX IT Services GmbH, Sentinel-2 cloudless (contains modified copernicus sentinel data 2017 and 2018) (https://s2maps.eu) (2018).
11. J West, M Lieber, S Lutter, H Schandl, Proposal for a new compilation system for metal ores in economy wide material flow accounting. *J. Ind. Ecol.* **24**, 1220–1233 (2020).
12. NT Nassar, TE Graedel, EM Harper, By-product metals are technologically essential but have problematic supply. *Sci. Adv.* **1**, e1400180 (2015).
13. MC Hansen, et al., High-resolution global maps of 21st-century forest cover change. *Science* **342**, 850–853 (2013).
14. D Baston, exactextractr: Fast extraction from raster datasets using polygons, r package version 0.5.1. (*The Comprehensive R Archive Network (CRAN)* https://CRAN.R-project.org/package=exactextractr) (2020).
15. NASA – Socioeconomic Data and Applications Center, Gridded population of the world, version 4 (gpwv4): Population density, revision 11 (*Center for International Earth Science Information Network, Columbia University* https://doi.org/10.7927/H49C6VHW) (2018).
16. UNEP, *Protected Planet: The World Database on Protected Areas 03/2020.* (UNEP-WCMC and IUCN, Cambridge), (2020).
17. ESA, Land cover (*Climate Change Initiative* https://maps.elie.ucl.ac.be/CCI/viewer/) (2020).
18. G Amatulli, et al., A suite of global, cross-scale topographic variables for environmental and biodiversity modeling. *Sci. Data* **5**, 180040 (2018).
19. G Amatulli, et al., A suite of global, cross-scale topographic variables for environmental and biodiversity modelling, links to files in geotiff format (*PANGAEA* https://doi.org/10.1594/PANGAEA.867115) (2018).
20. T Hengl, et al., Soilgrids250m: Global gridded soil information based on machine learning. *PLoS One* **12**, 1–40 (2017).
21. NH Batjes, E Ribeiro, A van Oostrum, Standardised soil profile data to support global mapping and modelling (wosis snapshot 2019). *Earth Syst. Sci. Data* **12**, 299–320 (2020).
22. ISRIC, Soilgrids (*World Soil Information* https://soilgrids.org/) (2016).
23. SM Iacus, G King, G Porro, Causal inference without balance checking: Coarsened exact matching. *Polit. analysis* **20**, 1–24 (2012).
24. SL Morgan, C Winship, *Counterfactuals and Causal Inference.* (Cambridge University Press), (2015).
25. HJ Geist, EF Lambin, Proximate causes and underlying driving forces of tropical deforestation. *BioScience* **52**, 143–150 (2002).
26. RS DeFries, T Rudel, M Uriarte, M Hansen, Deforestation driven by urban population growth and agricultural trade in the twenty-first century. *Nat. Geosci.* **3**, 178–181 (2010).
27. PG Curtis, CM Slay, NL Harris, A Tyukavina, MC Hansen, Classifying drivers of global forest loss. *Science* **361**, 1108–1111 (2018).
28. J Busch, K Ferretti-Gallon, What drives deforestation and what stops it? A meta-analysis. *Rev. Environ. Econ. Policy* **11**, 3–23 (2017).
29. TT Werner, et al., Global-scale remote sensing of mine areas and analysis of factors explaining their extent. *Glob. Environ. Chang.* **60**, 102007 (2020).
30. S Iacus, G King, G Porro, cem: Software for coarsened exact matching. *J. Stat. Software, Articles* **30**, 1–27 (2009).
31. G King, R Nielsen, Why propensity scores should not be used for matching. *Polit. Analysis* **27**, 435 (2019).
32. LJ Sonter, et al., Mining drives extensive deforestation in the Brazilian Amazon. *Nat. Commun.* **8**, 1013 (2017).
33. JE Ripollone, KF Huybrechts, KJ Rothman, RE Ferguson, JM Franklin, Evaluating the utility of coarsened exact matching for pharmacoepidemiology using real and simulated claims data. *Am. J. Epidemiol.* **189**, 613–622 (2020).
34. F Hayashi, Econometrics. *Princet. Univ. Press. Sect.* **1**, 60–69 (2000).
35. V Maus, et al., Global-scale mining polygons (version 1) (*PANGAEA* https://doi.org/10.1594/PANGAEA.910894) (2020).
36. N Greifer, *cobalt: Covariate Balance Tables and Plots*, (2020) R package version 4.2.4.
